# The processing of facial identity and expression is interactive, but dependent on task and experience

**DOI:** 10.3389/fnhum.2014.00920

**Published:** 2014-11-14

**Authors:** Alla Yankouskaya, Glyn W. Humphreys, Pia Rotshtein

**Affiliations:** ^1^Cognitive Neuropsychology Centre, Department of Experimental Psychology, University of OxfordOxford, UK; ^2^School of Psychology, University of BirminghamBirmingham, UK

**Keywords:** face processing, integration, identity, emotions, redundancy gains, capacity processing

## Abstract

Facial identity and emotional expression are two important sources of information for daily social interaction. However the link between these two aspects of face processing has been the focus of an unresolved debate for the past three decades. Three views have been advocated: (1) separate and parallel processing of identity and emotional expression signals derived from faces; (2) asymmetric processing with the computation of emotion in faces depending on facial identity coding but not vice versa; and (3) integrated processing of facial identity and emotion. We present studies with healthy participants that primarily apply methods from mathematical psychology, formally testing the relations between the processing of facial identity and emotion. Specifically, we focused on the “Garner” paradigm, the composite face effect and the divided attention tasks. We further ask whether the architecture of face-related processes is fixed or flexible and whether (and how) it can be shaped by experience. We conclude that formal methods of testing the relations between processes show that the processing of facial identity and expressions interact, and hence are not fully independent. We further demonstrate that the architecture of the relations depends on experience; where experience leads to higher degree of inter-dependence in the processing of identity and expressions. We propose that this change occurs as integrative processes are more efficient than parallel. Finally, we argue that the dynamic aspects of face processing need to be incorporated into theories in this field.

## Introduction

It is difficult to find more a complex source of information in social interaction than human faces. Gaze direction, emotional expression and identity are perceived very rapidly allowing us to make a judgment of a face seen for less than a hundred milliseconds. How is this broad range of facial information processed by our perceptual system? To answer this question, scientists have used two general approaches. The first focuses on the independent manipulation of each type of facial information, e.g., emotional expressions (Bassili, 1979; Bartlett et al., 1999; Baudouin et al., 2000; Calder et al., 2000; Adolphs, 2002; Balconi and Lucchiari, 2005); person identity (Bruce et al., 1991; Collishaw and Hole, 2000; Baudouin and Humphreys, 2006; Caharel et al., 2009). The second approach is to manipulate both types of information together, to determine whether different types of facial information are processed in an integrative or independent manner (Etcoff, 1984; Bruce and Young, 1986; Campbell et al., 1986; de Gelder et al., 2003; Wild-Wall, 2004; Calder and Young, 2005; Curby et al., 2012). The focus of this review is on studies adopting the latter approach to address the still outstanding question of whether identity and emotional expression information in faces are processed independently or interactively. We attempt to answer this question using novel application of mathematical procedures to psychological problems. We further discuss the novel hypothesis that the architecture of face processing is dynamic and shaped by experience.

Three paradigms are commonly used with healthy participants to assess the relationship between factors in systematic ways: the “Garner paradigm”, the facial composite paradigm and the divided attention paradigm. Methodological issues within each paradigm and the contrasting processes that they “weight” are described in detail. The review begins with a brief highlight of the three views on interactive vs. independent processing of identity and emotion in faces and the supporting evidence for each. The three following sections present the evidence on interactions between identity and emotional expression from studies employing each task. The last section summarizes our knowledge about the relations between identity and emotion processing in faces and proposes directions for further studies.

## Three views on interactions between identity and emotional expression processing in faces

A critical question, fundamental for building models of face processing, is whether identity and emotional expressions in faces interact or whether they are processed by strictly separated routes. This section provides a brief summary of contemporary views on the relationship between the two types of facial information. To date, three accounts have been proposed.

*The first account—independent processing—*proposes that there is separate and parallel processing of identity and emotional expression signals from faces (Bruce and Young, 1986). The main support for the separate-parallel routes comes from neuropsychological studies showing double dissociations in emotion and identity processing. Patients have been reported to have impaired recognition of face identity but not emotion (Bruyer et al., 1983; Jones and Tranel, 2001; Nunn et al., 2001), while other patients have impaired discrimination of face expression but not identity (Humphreys et al., 1993) or impairments at recognizing specific emotion (e.g., Adolphs et al., 1994; Calder et al., 2000).

*The second account—asymmetric dependency—*argues for asymmetric processing of identity and emotional expression in faces; namely that emotion processing depends on facial identity coding but not vice versa (Schweinberger and Soukup, 1998; Schweinberger et al., 1999; Baudouin et al., 2000; Kaufmann and Schweinberger, 2004; Atkinson et al., 2005). A common finding in studies that support asymmetric dependency is that observers are able to attend and respond to the identity of faces while ignoring emotional and speech expressions, but they are unable to ignore identity when attending and responding to either emotional expression or speech (Schweinberger and Soukup, 1998; Schweinberger et al., 1999). Similar results have been reported in studies examining the relationship between gender and emotion in faces (Le Gal and Bruce, 2002; Atkinson et al., 2005). These findings are consistent with the idea that information about invariant aspects of faces influences how changeable aspects of faces are computed, while information about their changeable aspects of faces does not influence the processing of invariant face properties (Haxby et al., 2000).

*The third account—interactive processing—*supports the idea of interactive processing between facial identity and emotion (Ganel and Goshen-Gottstein, 2002, 2004; Wild-Wall, 2004; Yankouskaya et al., 2012; Wang et al., 2013). Ganel and Goshen-Gottstein (2002, 2004) provide evidence for symmetric interference between facial identity and emotions in familiar faces and proposed that the mechanisms involved in processing familiar identity and expression are interconnected, with facial identity serving as a reference from which different expressions are more easily derived (Ganel and Goshen-Gottstein, 2002, 2004). Study by Yankouskaya et al. (2012) further support the interactive view by demonstrating redundancy gains and super capacity in processing faces containing both a target identity and emotional expression as compared when single target (a target identity or emotion) is present. The interactive model is also supported by neuroimaging findings (see for review Calder and Young, 2005).

It is important to note the asymmetric and symmetric interactive accounts do not necessarily imply that there is only one shared mechanism for processing identity and emotion information from faces (Calder and Young, 2005). These accounts suggest a high degree of interconnection between emotion and identity processing, whether they are incorporated in one representational space (Calder and Young, 2005), or in separate ones (Haxby et al., 2002).

In the following sections we discuss in detail evidence based on formal testing of the three models of identity and expression processing.

### The garner task

The Garner paradigm was originally designed to establish the nature of the relationship between the properties of two-dimensional stimuli (Garner, 1974). It is assumed that if two dimensions of a stimulus are processed interactively, variation in one dimension will interfere with processing of the second dimension. In contrast, if the two dimensions are processed independently, there will be no interference from each other. Typically an observer is required to make speeded two-choice classifications of four types of stimuli as the two dimensions of the stimuli are varied orthogonally. The stimuli are presented in three experimental conditions: a control condition (the stimuli vary along a relevant dimension, while the irrelevant dimension is held constant); an orthogonal condition (both the relevant and irrelevant dimensions vary); and a correlated condition (the two dimensions co-vary). Garner interference (GI) is defined as an increase in reaction times (RTs) and/or error rates for the relevant target dimension in the orthogonal condition relative to the constant and the correlated conditions. The difference between the correlated and constant blocks provides a measure for the potential benefit arising from integrating the two factors. Though this aspect is rarely considered in studies using the Garner paradigm.

Results based on the Garner paradigm provide conflicting results. While some studies show no interference in responses to either expression or identity, suggesting independent processing (e.g., Etcoff, 1984), others show an asymmetrical effect (effect of identity on expression but not vice versa; e.g., Schweinberger and Soukup, 1998), symmetrical effects with familiar faces (but not with unfamiliar faces) (e.g., Ganel and Goshen-Gottstein, 2004) or symmetrical interactions between facial expression and facial familiarity that emerge for some expressions (happiness and neutral), but not for others (disgust and fear) (Wild-Wall, 2004). One possible reason for the variability in the results may be the use of a small stimulus set in many studies using this paradigm. Typically only two different stimuli exemplars displaying one of two emotions are used (e.g., see Schweinberger and Soukup, 1998). This limited set of stimuli is repeated across trials allowing the development of a strategy of discriminating stimuli based on local image details (e.g., variations in lighting, photographic grain) rather than on expression and identity. Such a strategy may limit interference between the dimensions. Another important issue is that different picture-based strategies may be used for the identity and emotion decision tasks in the Garner paradigm. In the identity decision task pictorial strategies might be used to discriminate individuals based on the shape of a face or on non-facial cues such as hair style (e.g., see the stimuli in Etcoff (1984) and Schweinberger and Soukup (1998) for example). For the expression decision task however, where participants are required to attend to internal facial features, this strategy may be inappropriate. This can lead to differences in task difficulty which may contribute to the asymmetric interference effects between identity and emotional expression judgments.

The relative discriminability between the exemplars of the two dimensions can also affect results in the Garner paradigm. Wang et al. (2013) orthogonally manipulated the discriminability (Disc) of stimuli within the two relevant dimensions (e.g., high Disc identities and high Disc expressions, high Disc identities and low Disc expressions). The results showed asymmetric interference from identity to emotional expression when the discriminability of the facial expression was low and that of facial identity was high. In contrast there was interference from emotional expression on identity when the discriminability of facial expression was high and that of facial identity low. When both dimensions were low in discriminability, interference was found in both directions, while there was no interference when both dimensions were highly discriminable. The authors argued that, when discriminability is low, people refer to additional information from an irrelevant dimension, and this results in GI (Wang et al., 2013). Ganel and Goshen-Gottstein (2004) controlled for pictorial processing strategies and they also equated the discriminability of identity and expression judgments. In this case symmetric interference was found between expression and identity judgments, though only for familiar faces (Ganel and Goshen-Gottstein, 2004).

Taken together, the above studies suggest that degree of interaction between identity and emotional expression in faces is associated with the level of discriminability of the two dimensions. It is less clear, however, why no interaction is observed when both dimensions are highly discriminable. It is possible that participants process each relevant dimension separately from the irrelevant one, because there is enough information carried by each dimension. However, there is also the possibility that in the orthogonal condition participants tend to switch their attention between the two dimensions that constantly change. Hence in some occasion participants direct attention to the irrelevant dimension which leads to potential increase in errors and longer RT. Thus, the effects of the unattended stimulus dimensions arise due to trial-by-trial fluctuations in attention that lead to the irrelevant dimension sometimes being attended (Lavie and Tsal, 1994; Weissman et al., 2009). On these occasions performance will be affected by variation in the irrelevant dimension, even though the dimensions might be processed independently.

### The composite face task

Composite faces combine the top half of one face with the bottom half of another face. When aligned, the two face halves appear to fuse together to produce a novel face, making it difficult to selectively process either half of the composite by itself (Young et al., 1987; Mondloch et al., 2006; Rhodes et al., 2006; McKone, 2008; Rossion, 2013). In the composite paradigm, the task is to attend to one half of the face (e.g., the top), and either name it (naming version) or determine whether it is the same or different to the half face in a second composite stimulus (matching version), while ignoring the non-target half (e.g., the bottom part of the face). There are two critical conditions: when the two halves of the faces are aligned—“encouraging” holistic processing, or when the two halves are not aligned—when there is less likelihood of processing them as a single perceptual unit. Note, that as in the Garner paradigm, perceptual integration is indexed by the level of interference of the irrelevant dimension on the processing of the relevant dimension.

When the two halves of the faces are smoothly aligned, the novel face in the composite condition can create a conflicting situation as it does not match the identity of either the top or the bottom half. In contrast, when two halves are misaligned, the face is not encoded as a perceptual whole, and the information of either part can be assessed without mutual interference. The robust finding is that participants are slower, and less accurate in identity judgments of the top half when the face halves are vertically aligned compared to when they are spatially unaligned (e.g., Young et al., 1987; McKone, 2008). Similar to the effects with facial identity, there is also a composite effect for emotional expressions (Calder et al., 2000, Experiment 1).

Interestingly, when identity and expression information are combined, the composite effect in identity has been found to operate independently of the effect in emotional expression. In (Calder et al., 2000, Experiment 4), three types of composite faces were employed: (i) two halves of the same person posing different facial expressions (same-identity/different-expression composites); (ii) two halves of different people posing the same facial expression (different-identity/same-expression composites); and (iii) two halves of different identities posing different facial expressions (different-identity/different- expression composites). Participants performed two tasks: judging the identity or the expression of each face. The RT pattern depended on the task. In the identity task, judging the identity of the top half of the face was facilitated if it matched the identity of the bottom half, and this was independent of whether the expressions (the irrelevant dimension in this case) matched or mismatched. Similarly in the expression task, when the two halves were matched for expression responses were facilitated independent of facial identities. Thus, the results indicated that people could selectively attend to either of the facial dimensions (see a similar conclusion in Etcoff’s (1984) study where participants performed a Garner task).

Critical examination of Calder et al.’s (2000) Experiment 4 highlights a few important points. First the authors did not equate for difficulty across the condition and trial types (e.g., identity decisions were easier than expression decisions). It could be that when decisions are easier, participants tend to rely on a single source of information to make the decision (Wang et al., 2013); however if the decision is difficult the participants may refer to the irrelevant dimensions to provide additional information to make a correct classification judgment or they may need a longer time to ignore the irrelevant information. In both cases this does not imply complete independence between the coding of identity and emotional expression. Second, the high cognitive demands on the perceptual system, required to focus attention on just one part of the faces, may have affected the results. For example, similar to the Garner task, participants may have attended to the irrelevant dimension due to trial-by-trial fluctuations in attention or local details of the images. Finally, the results may reflect a tradeoff between speed and accuracy, as the accuracy results indicate that most errors were made during conditions where the top and bottom halves did not match on either expression or identity. Furthermore, Richler et al. (2008) found that discriminability (*d’*) on trials when both face halves had same identity was higher than discriminability on trials when the two halves had different identities. In summary, the composite face task cannot unambiguously provide evidence for separate routes for processing of facial identity and emotional expressions.

### The divided attention task

The divided attention task has been used in studies examining holistic vs. featural processing in faces (Wenger and Townsend, 2001) and independent vs. interactive processing of identity and expressed emotion in faces (Wenger and Townsend, 2001; Yankouskaya et al., 2012, 2014a,b).

In the divided attention task, participants are required to monitor two sources of information simultaneously for a target to decide if the target is present or absent. There are two main advantages in employing the divided attention task. First, the task requires people to attend to facial identity and emotional expression simultaneously—a situation that closely resembles daily life. Second, in contrast to the selective attention task, the divided attention task controls for performance in the single target conditions by including the double target display. There is considerable evidence that, when a visual display contains two targets that require the same response, RTs are faster compared to when only one target appears (Miller, 1982; Mordkoff and Miller, 1993; Miller et al., 2001; Wenger and Townsend, 2006). For example, in Mordkoff and Miller’s (1993) study participants were required to divide their attention between the separable dimensions of color and shape, with all stimulus features being attributes of a single object. Participants were asked to press a button if the target color (green), the target shape (X), or both target features (green X) were displayed, or to withhold their response. The mean RT on redundant target trials was significantly less than the mean RT on single target trials (Mordkoff and Miller, 1993).

Although different explanations can be put forward to account for this redundant target effect (RTE), the most relevant here are the Independent Race Model (Raab, 1962) and the Coactivation Model (Miller, 1982). According to the Independent Race Model, redundancy gains are explained by means of “statistical facilitation” (Raab, 1962). Whenever two targets are presented simultaneously, the faster signal determines the response “target present” (i.e., this signal wins the race). As long as the processing time distributions for the two signals overlap, RTs will be speeded when two targets occur since the winning signal can always be used for the response (Raab, 1962). Note, that signal which finishes “first” may depend on whether it is attended. For example, emotional expression or identity may be computed first, if there are fluctuations in attention to each independent dimension.

An alternative explanation for the RTE is the coactivation view. According to this model, the information supporting a response “target present” is pooled across the features defining the targets prior to response execution (Miller, 1982). When both target identity and target emotional expression contribute activation toward the same decision threshold, the response will be activated more rapidly relative to when only one attribute contributes activation.

The critical contrast for the two models compares the probability for the response times obtained on redundant targets trials relative to the sum of probabilities for responses being made to either single target trial. The Independent Race Model holds that at no point in the cumulative distribution functions should the probability of a response to redundant targets exceed the sum of the probabilities for responses to either single target. In contrast, the coactivation account predicts that responses to the redundant targets can be made before either single target generates enough activation to produce a response. Thus, the number of fastest responses to a face containing both the target identity and the target emotional expression should be larger than the number of fastest responses to either target facial identity or target expression when presented as single targets. The procedure assessing the relations between the number of fast responses in the single target trials vs. the dual target trails is referred to as the Miller inequality test, or the race model inequality test.

An alternative approach to test independence vs. co-activation processing is by examining the effects of the RTE on the workload capacity of the system (Townsend and Nozawa, 1995). The concept of workload capacity reflects the efficiency with which a cognitive system performs a task. Mathematically, the workload capacity (*C(t)*) is defined by the hazard function that gives the rate of process completion at any point time (when the process under an observation has not yet completed) (Townsend and Wenger, 2004). Importantly, the yardstick for the capacity model (Townsend and Nozawa, 1995) is the standard parallel model (e.g., The Independent Race Model (Raab, 1962)) where processing on individual dimensions does not change with increasing workload and signals are processed in parallel without mutual interference. In terms of the capacity model, the standard parallel processing model is associated with unlimited capacity (*C(t)* = 1), as processing one dimension has no impact on the processing of the second dimension. Processing with limited capacity (*C(t)* < 1) is associated with decreasing performance (e.g., slowing in RT) when the workload increases and the system performs sub-optimally. On the other hand the overall workload could decrease when redundant targets are presented, leading to facilitation in performance (e.g., faster RT). In this case the system is said to operate at super capacity (*C(t)* > 1)). The super capacity emerges since a decision is made before any single dimension alone provides sufficient evidence to support it. Hence less processing was needed of each dimension to enable a decision—making the process more efficient. The super capacity mode violates the race model inequality (Townsend and Wenger, 2004; Townsend and Eidels, 2011), suggesting positive dependency between the two dimensions.

**Figure 1:**
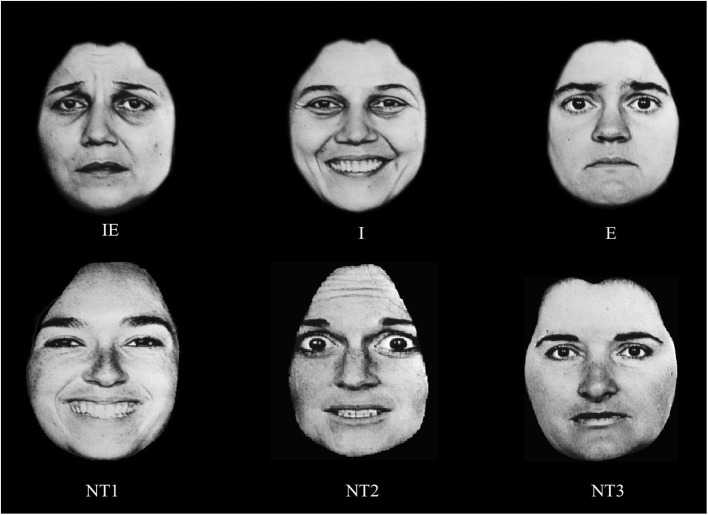
**An example of the stimuli in Yankouskaya et al. (2012)**. IE—a face containing both the target identity and the target emotional expression; I—a face containing the target identity but not the expression; E—a face containing target emotional expression; NT1–NT3 faces containing neither the target identity nor the target emotion. In this study we used faces from the NimStim database, but because of publication restriction on faces from that database, we presenting here other faces (taken from Ekman, 1993) as examples only.

The Race Model and the capacity measure have been used in tests of independence vs. coactivation in the processing of facial identity and emotional expression. Yankouskaya et al. (2012) employed the divided attention task under conditions where participants had to detect target identities and target emotional expressions from photographs of a set target faces. Three of these photographs contained targets: stimulus 1 had both the target identity and the target emotion (i.e., redundant target); stimulus 2 contained the target identity and a non-target emotional expression; stimulus 3 contained the target emotional expression and a non-target identity (Figure 1). Three non-target faces were photographs of three different people, and expressed emotions different to those in target faces. Identity, gender and emotional expression information were varied across these studies.

The general results showed that supper-additive redundancy gains occurred between face identity and emotional expression. Particularly striking was the finding that there were violations of the race model inequality test (Miller, 1982) when the target identity was combined with the target expression in a single face. Violation of the race model inequality occurred for combinations of sad or an anger expression with facial identity but not when identity was combined with a neutral expression. In the last case, the authors report no evidence for any redundancy gain. Yankouskaya et al. (2012) suggest that unfamiliar faces bearing a neutral expression do not carry expression-contingent features and a neutral expression may be defined by the absence of an expression, making it more idiosyncratic to the particular face.

Importantly, the mathematical tests of the race model and capacity measures provide us with a precise analysis of the relationship between the processing of identity and emotional expression (Yankouskaya et al., 2012), facilitating estimation of the effect of different factors on the relationship (Yankouskaya et al., 2014a,b).

Taken together the data derived from the divided attention task within the framework of the race model and capacity measures of processing are consistent with coactive processing when a target identity is paired with a distinct emotional expression. The coactivation is beneficial for the cognitive system as it allows to pool together information derived from identity and emotion in faces leading to super capacity of the system. This super capacity emerges since combining information reduces the demands of resources compared to when each channel is consider independently.

## Do experience and familiarity with faces modulate the way that expression and identity processing interact?

Based on common observation, the recognition of identity and emotional expression in faces in everyday life is easy. We can catch a face of familiar person in a crowd or an expression in a face in few seconds. In return, we are typically quick at making a judgment if a briefly seen face is unfamiliar or whether a stranger’s face has a particular expression. On the other hand, it may take longer for us to recognize a familiar face with an unusual expression or a stranger’s smiling face, because it makes us doubt whether the person is familiar or not (Baudouin et al., 2000). These examples show that familiarity judgments to faces are affected by the expression of the faces, and the interaction occurs for both unfamiliar and familiar faces (Baudouin et al., 2000; Elfenbein and Ambady, 2002; Eastwood et al., 2003; Wild-Wall, 2004; Calvo and Nummenmaa, 2008). Familiarity with faces can be conceptualized at multiple levels: (1) continuous contact across the lifespan with faces in general may gradually shape the way we process faces; (2) there may be familiarity for faces from specific ethnical/relevant cultural group; and (3) there may be familiarity and increased experience with the face of specific individuals (including both media channels and direct social interactions).

Experience with human faces changes across the lifespan and affects the way we process faces. For example, the processing of both identity and expressions improves from childhood to adulthood (Schwarzer, 2000; Baudouin et al., 2010; Germine et al., 2011) and gradually declines in older people (Plude and Hoyer, 1986; Ruffman et al., 2008; Obermeyer et al., 2012). It is unclear, however, whether general experience with faces through the lifespan affects the way identity and expression interact.

**Figure 2:**
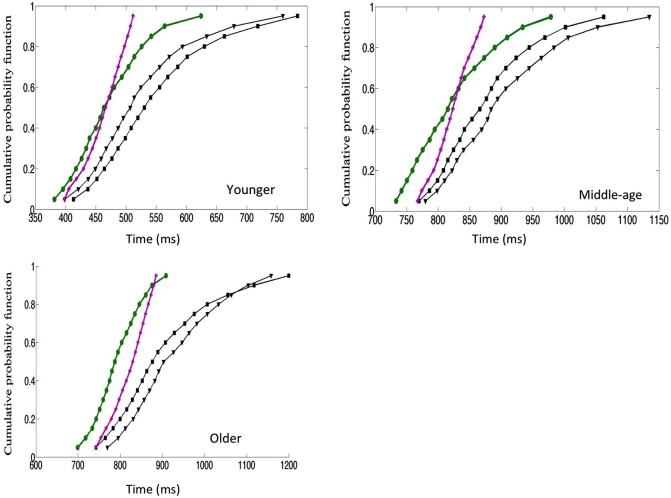
**Cumulative distribution function plots (CDFs)^1^**. The *x*-axis presented the RTs, the *y*-axis present CDF. For a given point on the CDF the total number of trials in each condition (value on *y*) with RT less than specified value on the *x*-axis. The redundant targets (IE) are plotted in green, the sum of the distributions of the single targets: emotional expression and identity targets (I + E) is plotted in purple and each single targets (E) and (I) is plotted in black. The crucial comparison is between the green and the purple lines. Results for the young are presented in the top left panel, middle aged in top right panel and older in the lower panel (data reported in Yankouskaya et al., 2014b).

**Figure 3:**
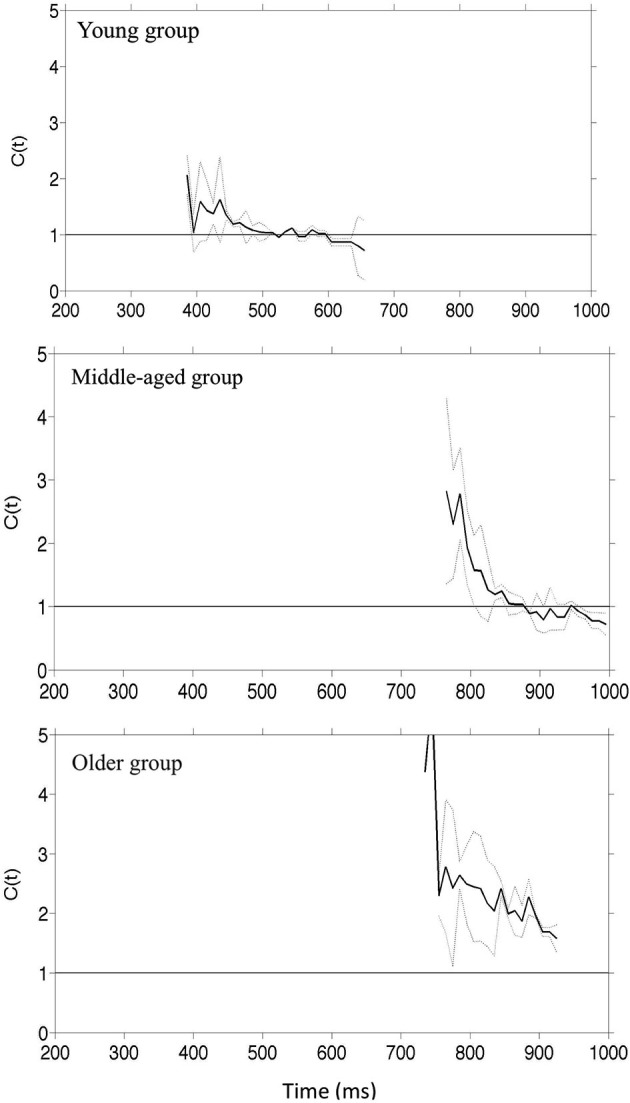
**Capacity coefficients for the three groups of participants: top row young adult, middle row–middle-aged people**. The horizontal line at *C(t)* = 1 indicates the reference value for unlimited capacity. The capacity coefficients are depicted in solid line; the confidence interval is in dashed line (data reported in Yankouskaya et al., 2014b).

We used the divided attention paradigm to assess how aging affects the integration of visual information from faces. Three groups of participants aged 20–30, 40–50 and 60–70 performed a divided attention task in which they had to detect the presence of a target facial identity or a target facial expression. Three target stimuli were used: (1) with the target identity but not the target expression; (2) with the target expression but not the target identity; and (3) with both the target identity and target expressions (the redundant target condition). On non-target trials the faces contained neither the target identity nor the target expression. All groups were faster in responding to a face containing both the target identity and emotion compared to faces containing either single target. Furthermore the redundancy gains for combined targets exceeded performance limits predicted by the independent processing of facial identity and emotion. These results held across the age range suggesting that there is interactive processing of facial identity and emotion which is independent of the effects of cognitive aging. Remarkably, there was an increase in the extent of co-activation across trials throughout the adulthood lifespan so that, with increased age the benefits of redundant targets were larger. This was reflected by an increased probability of fast response trials and increased processing efficiency evidenced by “higher” super-capacity. (Figures 2, 3).

**Figure 4:**
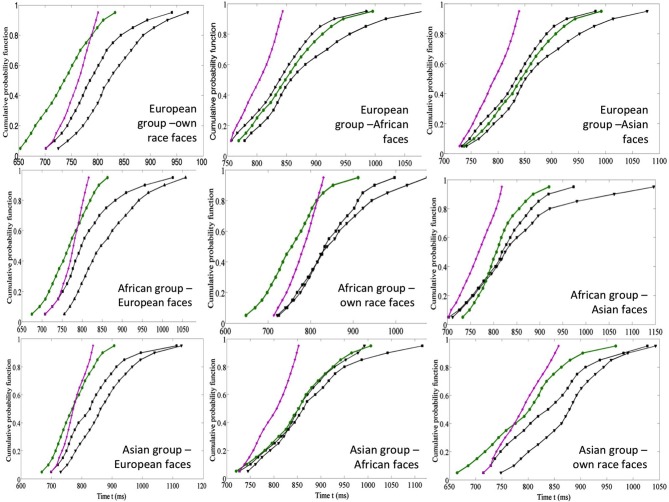
**Data from the race inequality test for three groups of participants: European, African and Asian: top row European participants (own-race, African and Asian faces from the left to the right), middle row–African participants (European, African and Asian faces from the left to the right), low row–Asian participants (European, African and own-race faces from the left to the right)**. I—target identity and E—target emotion (in black), IE—both target identity and target emotion (in green), I + E—the sum of distributions for I and E (in purple). These graphs show whether the redundant target information is processed coactively (IE line places on the left of the I + E line, see for details Yankouskaya et al., 2014a).

The evidence on the effects of life experience with faces is mirrored by the data on processing faces from same vs. a different race. It is well documented that the processing of own-race faces is advantaged for both expressions (Elfenbein and Ambady, 2002; Kubota and Ito, 2007) and identity (Levin, 2000; Kito and Lee, 2002; Walker and Tanaka, 2003; Michel et al., 2006; Cassidy et al., 2011). In a recent study Yankouskaya et al. (2014a) showed that experience with own race faces affected the integration of identity and emotional information. The relations between the processing of facial identity and emotion in own- and other-race faces were examined using a fully crossed design with participants from three different ethnicities all residing in the UK at the time of the study (Yankouskaya et al., 2014a). Three groups of participants (European, African and Asian individuals) performed the divided attention task on three sets of six female portrait photographs for each ethnic group. In each set, three photographs contained targets: Stimulus 1 had both the target identity and the target emotion, sad (IE); Stimulus 2 contained the target identity and a non-target emotional expression, happy (I); Stimulus 3 contained the target emotional expression, sad, and a non-target identity (E). Three non-target faces were photographs of three other people expressing emotions different from those in target faces (angry, surprised, and neutral). The benefits of redundant identity and emotion signals were evaluated and formally tested in relation to models of independent and coactive feature processing and measures of processing capacity for the different types of stimuli (see details in section 1.3). The results suggested that coactive processing of identity and emotion that was linked to super capacity for own-race but not for other-race faces (Figure 4).

Furthermore, in the study of Yankouskaya et al. (2014a), the evidence for a race effect on the integration of emotion and identity information was asymmetric. European participants only showed evidence of perceptual integration for their own race faces. However African and Asian participants showed this both for their own race faces and for European faces, but they did not show it respectively for Asian and African (both other-race) faces (Figure 4). This asymmetry reflects number of contacts with other race faces; as all participants were residing in the UK at the time of testing, the Asian and African participants had greater familiarity with European faces than Europeans had with Asian and African faces (Table 1). A formal test show that variations in the size of the redundancy gains across other race faces were strongly linked to the number of social contacts, but less so to the quality of the contact with other-race members. This suggests that experience with faces facilitates the coactive processing of identity and emotional expression.

**Table 1:** **Mean number (standard deviation in brackets) of well-known own and other-race people for groups of European, African and Asian participants**.

Group of participants	Number of well-known own and other race people		
	European	African	Asian
European	6.8* (2.1)	3.2 (1.3)	2.9 (0.6)
African	9.3 (3.4)	**16.7 (4.1)**	7.8 (4.2)
Asian	5.1 (2.2)	5.3 (2.5)	**11.4 (4.9)**

* In bold for own race people.

**Figure 5:**
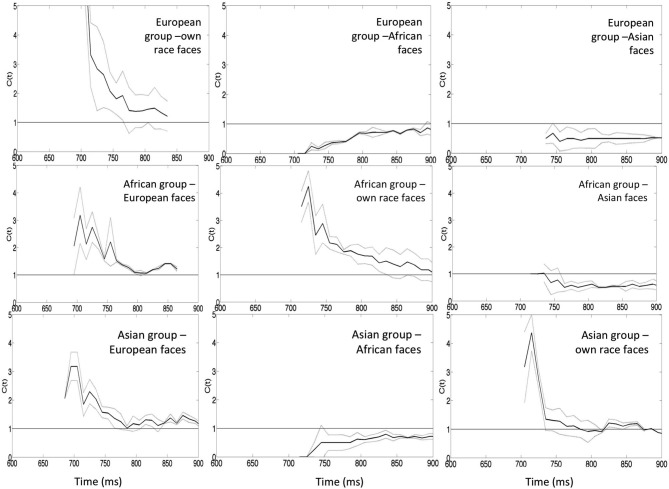
**Capacity coefficients for the three participants: top row European participants (own-race, African and Asian faces from the left to the right), middle row–African participants (European, African and Asian faces from the left to the right), the bottom row—Asian participants (European, African and Asian faces from the left to the right)**. The horizontal line at *C(t)* = 1 indicates the reference value for unlimited capacity. The capacity coefficients are depicted in solid line; the confidence interval is in dashed line. Data reported in Yankouskaya et al. (2014a).

The capacity analysis also demonstrated super capacity for processing identity and emotional expression within own-race faces, indicating that the observed responses for the redundant target face were greater than predicted by the combined response to single targets (Figure 5). In contrast, adding information to other-race faces generated results indicative of a negative dependency and suggesting that the processing of identity and emotional expression in other-race faces operates with limited capacity. The negative dependency for other-race faces held true for European participants but not for African and Asian groups where responses for European faces showed positive dependency.

Collectively, these results suggest that one component of the own race face advantage is the increase in the integration of identity and emotional expression information in own-race faces. This effect is strongly linked to individual experience with particular types of face.

Finally, familiarity with specific individuals can also change the way information from the face is processed. Ganel and Goshen-Gottstein (2004) predicted that GI should be greater for familiar compared to unfamiliar faces, because representations of familiar faces contain richer and more detailed structural descriptions than representations of unfamiliar faces. As a consequence perceivers should be more likely to be sensitive to the associations between invariant and changeable aspects of familiar faces than they are to those of unfamiliar faces (Ganel and Goshen-Gottstein, 2004). This was demonstrated using the Garner paradigm where participants had to make identity and emotion judgments for personally familiar and unfamiliar faces. The authors report that interference between identity and expression increased for familiar faces (Ganel and Goshen-Gottstein, 2004), consistent with this information being processed in a more integral way in this case.

Taken together, the studies above suggest that familiarity modulates the relationship between the processing of identity and emotional expression in faces. Increased experience with faces lead to increased integration of information. As discussed above, pooling information across multiple channels allow the system to operate at super capacity, so enhancing processing efficiency. We suggest that experience with faces results in a qualitative change to the way faces are processed. Importantly this change occurs in adulthood, demonstrating that our face processing system retains flexibility throughout life. Furthermore, the above results show that there is no one system for processing faces, but multiple mechanisms operate in parallel depending on the faces processed and on our previous experience with them—for example, the identity and emotion of novel faces (e.g., faces from a different ethnicity) are processed in parallel, while identity and emotion information from highly familiar face types are integrated. Thus we propose that experience shapes the connections between different processing channels and thereby increasing the efficacy of the processing in each of the individual channels. This brings up the question at what stage of the face processing identity and emotions are connected.

## At what stage of the processing information on identity and emotion is integrated

There are several stages of processing at which identity and expression/emotion could interact during face processing. The coactivation view (Miller, 1982) suggests that the interaction between identity and emotional expression leading to a super-redundancy gain occurs just after the two stimuli have been separately coded, but prior to a decision about target presence. The interactive view (Mordkoff and Yantis, 1991) suggests that information about facial identity and emotional expression may be exchanged at early perceptual levels (inter-stimulus crosstalk) or at a decisional stage (non-target response bias). We next briefly discuss studies which may offer some resolution to these conflicting views.

Evidence for separate mechanisms for emotion and identity processing that interact prior to the decision comes primarily from neuropsychological cases and neuroimaging studies. The neuropsychological evidence mentioned above (Behrmann et al., 2007; Riddoch et al., 2008) showing a double dissociation between expression and identity processing. Neuroimaging studies suggest that different neural structures are involved in processing identity (invariant) and emotion (variant) information (Haxby et al., 2002). For example, it is shown that regions within the superior temporal process expressions, while regions along the Fusiform Gyrus process identity (Winston et al., 2004). It is further shown that processing within these two regions is relatively separated (Fairhall and Ishai, 2007). Taken together it is suggested that at some stage identity and expression are processed separately.

The alternative view suggests a single mechanism for processing identity and expressions from faces (Calder and Young, 2005). Thus arguing that identity and expression are not processed by dissociated mechanisms, but instead these two dimensions are processed within a single multi-dimensional space. This view relies on computational, neuropsychological and neuroimaging evidence. Computationally, it is shown that the principle components derived from pictures of different identity posing different expressions, contains identity specific, emotion specific and shared emotion and identity components (Cottrell et al., 2002). Thus the authors argue that within a single face representation system, different dimensions code for dissociated as well as shared features across the two dimensions. Critical review of neuropsychological studies by Calder and Young (2005) further suggest that most patients who are impaired at identity processing (prosopagnosia) also show impaired emotion recognition, when formally tested, albeit less severe. Finally, Calder and Young review neuroimaging studies showing that regions along the Fusiform Gyrus (assumed to be solely processing identity) often show sensitivity to the facial expression (Vuilleumier et al., 2003) while regions along the superior temporal (assume to be dedicated to expression) are often sensitive to the face identity (Winston et al., 2004).

In summary, it is unclear whether the interactive nature of emotion and identity arise from a single multi-dimensional space or due to interaction between different processing streams. Further research is needed to address this question, maybe using methods that have higher time resolution such as EEG or MEG.

## Conclusion

We started our review by outlining three accounts for the relationship between the processing of identity and emotional expression in faces: independent, asymmetric and co-active processing of the two facial dimensions. We discussed in details support for each account from studies employing the Garner inference paradigm, the composite faces paradigm, and the divided attention paradigm. Based on this we conclude:

First, there is compelling evidence against strictly independent processing of identity and emotional expression (Ganel and Goshen-Gottstein, 2002, 2004; Wang et al., 2013), with perhaps the strongest evidence coming from studies of redundancy gains (particularly the mathematical tests against models assuming independent processing of expression and identity) (Yankouskaya et al., 2012, 2014a,b; Fitousi and Wenger, 2013).

Second, there are two crucial conditions for the interaction to occur: equal discriminability of identity and emotional expression (Ganel and Goshen-Gottstein, 2002; Wang et al., 2013) and an expression that is emotionally valenced (i.e., other than a neutral expression) (Yankouskaya et al., 2012).

Third, interactive processing of identity and emotional information in faces is modulated by familiarity and experience with faces (Ganel and Goshen-Gottstein, 2002; Yankouskaya et al., 2014a). Both greater familiarity and experience with faces facilitate the interaction.

## Conflict of interest statement

The authors declare that the research was conducted in the absence of any commercial or financial relationships that could be construed as a potential conflict of interest.

## Footnotes

^1^Graphic representations of the distributions were constructed using group RT distributions obtained by averaging individual RT distributions (Ulrich et al., 2007). When the CDFs are plotted, the Independent Race Model requires that the CDF of the redundant targets trials falls below and to the right of the summed CDF (less fast responding trials for the redundant target compared with the number of fast trials for both single targets), any reliable violation of this pattern provides support for the co-activation model.
